# *BDNF* Val66Met Polymorphism, the Allele-Specific Analysis by qRT-PCR - a Novel Protocol

**DOI:** 10.7150/ijms.50643

**Published:** 2020-10-18

**Authors:** Gilmara Gomes de Assis, Jay R. Hoffman, Eugene V. Gasanov

**Affiliations:** 1 Gdansk University of Physical Education and Sport, Faculty of Physical Education, Gdansk, Poland;; 2 Mossakowski Medical Research Centre, Polish Academy of Sciences, Warsaw, Poland;; 3 Department of Physical Therapy, Ariel University, Ariel, Israel.; 4 International Institute of Molecular and Cell Biology in Warsaw, Poland.

**Keywords:** Brain-derived Neurotrophic Factor, Allele-specific detection, ddRT-PCR, Gene expression, Exercise metabolism, Reference gene

## Abstract

**Background:** Alteration in brain-derived neurotrophic factor (BDNF) production is a marker of neuropathological conditions, which has led to the investigation of Val66Met polymorphism occurring in the human BDNF gene (*BDNF*). Presently, there are no reported methods available for the analysis of Val66Met impact on human *BDNF* functioning.

**Purpose:** To develop a qRT-PCR protocol for the allele-specific expression evaluation of the Val66Met polymorphism in *BDNF*.

**Methods:** Using RNA extracted from muscle samples of 9 healthy volunteers (32.9 ± 10.3 y) at rest and following a maximal effort aerobic capacity exercise test, a protocol was developed for the detection of Val66/Met66 allele-specific *BDNF* expression in Real-Time Quantitative Reverse Transcription PCR (qRT-PCR) - relative to housekeeping genes - and validated by absolute quantification in Droplet Digital Polymerase Chain Reaction (ddPCR).

**Results:** Differences in the relative values of BDNF mRNA were confirmed by ddPCR analysis. *HPRT1* and *B2M* were the most stable genes expressed in muscle tissue among different metabolic conditions, while *GAPDH* revealed to be metabolic responsive.

**Conclusion**: Our qRT-PCR protocol successfully determines the allele-specific detection and changes in *BDNF* expression regarding the Val66Met polymorphism.

## Introduction

Brain-derived neurotrophic factor (BDNF) is a bioactive protein from the family of neurotrophins, neurotrophic growth factors, with the most abundant expression in the neural system development and maturity throughout the mammalian lifespan 1,2. With compulsory roles in neurogenesis, synaptogenesis, neuronal repair and protection, alteration in BDNF dynamics may result in various neurological disorders and neurodegenerative diseases 3,4,5,6.

Predominantly expressed in neural and skeletal muscle cells as a protein signaler 7, BDNF synthesis results in the formation of several precursor isoforms in a multistage process involving cleavage and storage 2,8. The BDNF gene (*BDNF*) transcript encodes a precursor protein (pre-pro-BDNF isoform) which loses its secretory signal peptide (pre-region) and dimer into the pro-BDNF isoform. Pro-BDNF is subsequently cleaved by proprotein convertases in either the Golgi apparatus or in secretory vesicles to form mature BDNF 2,9. Released BDNF acts locally via both autocrine and paracrine mechanisms 10,11.

A single nucleotide polymorphism (SNP) has been identified in the human *BDNF* gene, resulting in a valine (Val) for methionine (Met) substitution in the 66th position of pre-pro-BDNF (Val66Met). This SNP in the *BDNF* gene is thought to have important implications for the gene and protein functioning. The *BDNF* Val66Met has been suggested to impair activity dependent release of BDNF 12, play a role in BDNF/pro-BDNF secretion ratios 13, and is also associated with increased susceptibility to cognitive deficits and neuropsychiatric disorders 14,15. Implications for the Met66-allele presence have been evidenced in experimental models 16;17 in which the complexity of the post-translational mechanisms was unable to determine impairments in the neurotrophin's function 18. In a clinical perspective, circulating BDNF concentration is likely to be associated with a greater acute response of cortisol to stress, especially in individuals with a psychiatric disorder 19. Although circulating blood concentrations of BDNF may provide some indication of BDNF production 20, it provides little understanding of the *BDNF* Val66Met polymorphism expression.

The differential expression of *BDNF* Val66Met polymorphic alleles to physical exercise was first detected in a recent study 21. Most clinical studies have reported changes in circulating BDNF concentrations during aerobic activities 22, but inconsistent in regards to resistance training 23. Increases in BDNF concentrations appear to be associated with the intensity of aerobic exercise 24,25, but may decrease during the recovery period if the exercise stimulus created a significant elevation in oxidative stress 20. Considering the increasing interest in the Val66Met polymorphism 26,27, and its possible implications in a variety of neuropathological processes, the purpose of this study was to determine the validity of assessing human* BDNF* expression *in vivo* by developing a Real-Time Quantitative Reverse Transcription Polymerase Chain Reaction (qRT-PCR) protocol using a standard maximum aerobic capacity test. Based upon the more consistent response of BDNF to higher intensity endurance exercise protocols, we believed that this protocol would best provide for the differential analysis of *BDNF* expression regarding the Val66Met polymorphism validated by absolute quantification in QX200™ Droplet Digital™ Polymerase Chain Reaction (ddPCR).

## Materials and methods

### Participants

Nine apparently healthy and physically active volunteers participating in medium and long distance Polish race circuits (10 and 21 km), with known genotypes for the *BDNF* gene agreed to participate in this study. Descriptive characteristics for all participants can be observed in Table 1. After an explanation of all procedures, risks, and benefits, each participant completed a Par-Q questionnaire 28 and provided his or her written informed consent to participate in the study. This study was conducted in accordance to Regulation 2016/679 of the European Union Parliament Council of 27 April 2016 on the protection of individuals with regard to the processing of personal data and on the free movement of such data, and repealing Directive 95/46 / EC (general data protection regulations) approved by the Ethical Committee of the Central Clinic Hospital of the MSWiA in Warsaw, Poland.

### Experimental Design

All study participants reported to the Human Performance Laboratory in the Mossakowski Medical Research Centre, Polish Academy of Science, Warsaw, Poland. Prior to the testing session, participants reported to the laboratory 2-hours post-prandial and were instructed not to consume any caffeine or alcohol; they were also instructed not to perform any lower body physical activity within 48 hours of testing. During the visit, all participants were provided with a brief medical exam that included anthropometric measurements. In addition, two muscle biopsies were obtained from participants prior to, and following performance of a maximal aerobic capacity test.

### Muscle Biopsies

Participants were required to rest in a supine position for 30 min. A muscle biopsy (50 mg wet weight) from the middle portion of the *Vastus lateralis* was obtained by a medical doctor, using local anesthesia and a semi-automatic 14-gauge biopsy needle (Guillotine needle double shoot DSGBL 14/10, Tsunami, Italy). Following the first sampling, participants were required to perform a maximal aerobic capacity test. Following the conclusion (within 1- 2 min) of the aerobic capacity test, a second muscle sample was obtained. Muscle samples were immediately placed in dry ice before storage at -80° C. All tests were performed between 3 p.m. and 5 p.m. (GMT+2).

### Maximal Aerobic Capacity Test

The maximal aerobic capacity test was conducted using the standard Bruce Protocol 29. The treadmill protocol began with each participant performing a 3-min walk at 2.7 km·h^-1^ and at a 10% incline. A progressive increase in both speed and inclination occurred every 3-min (stages), until participant's volitional fatigue. VO_2_max (maximum rate of oxygen consumption) was determined to be the highest 30-s VO_2_ value recorded during the test and coincided with at least two of the following three criteria: (a) 90% of age-predicted maximum heart rate; (b) respiratory exchange ratio > 1.1; and/or (c) a plateau of oxygen uptake (less than 150 mL/min increase in VO_2_ during the last 60 s of the test). Ventilation parameters were monitored and recorded from rest and throughout the protocol. Heart rate (bpm) and respiratory gases were assessed using Vmax29-Sensor Medics (CareFusion, USA) gas analyzer, and CardioSys software, version 4.1 (Marquette Hellige GmbH, Germany) (Data are presented in Table 1).

### Muscle RNA extraction

The muscle samples (50 mg) were manually homogenized in Eppendorf tubes with 0.5 ml of TRIzol reagent (T9424, Sigma-Aldrich, USA) and TRIzol-chlorophorm RNA extraction was performed according to the manufacturer's protocol. The total RNA was dissolved in 0.03 ml of RNAse-free water and concentrations were determined by NanoDrop™ 2000 Spectrophotometer (Thermo Scientific, USA). RNA samples were stored at -80°C. One µg of RNA was used for cDNA synthesis in 0.02 ml of reaction mixture using the iScript™ Reverse Transcription kit (BioRad, USA) according to the manufacturer's protocol. Samples of 1 and 3 μL of this cDNA solution was used as a template for qRT-PCR and ddPCR analyses.

### Reference Gene Establishment

To quantify BDNF mRNA expression in muscle we first established a gene of reference - which is expressed in a constant and stable rate both during homeostasis and stressed (e.g. exercise) conditions - i.e., housekeeping gene. To select the most applicable reference gene we used the muscle samples of three participants from the set, at both resting and post-exercise conditions, and performed a qRT-PCR analysis using the BioRad Prime PCR Pathway assay and Reference gene panel, and SsoAdvanced™ Universal SYBR® Green Supermix. Participant samples from both sexes with a maximal discrepancy in age were used for the reference gene establishment in order to address the highest heterogeneity.

**Figure 1:**
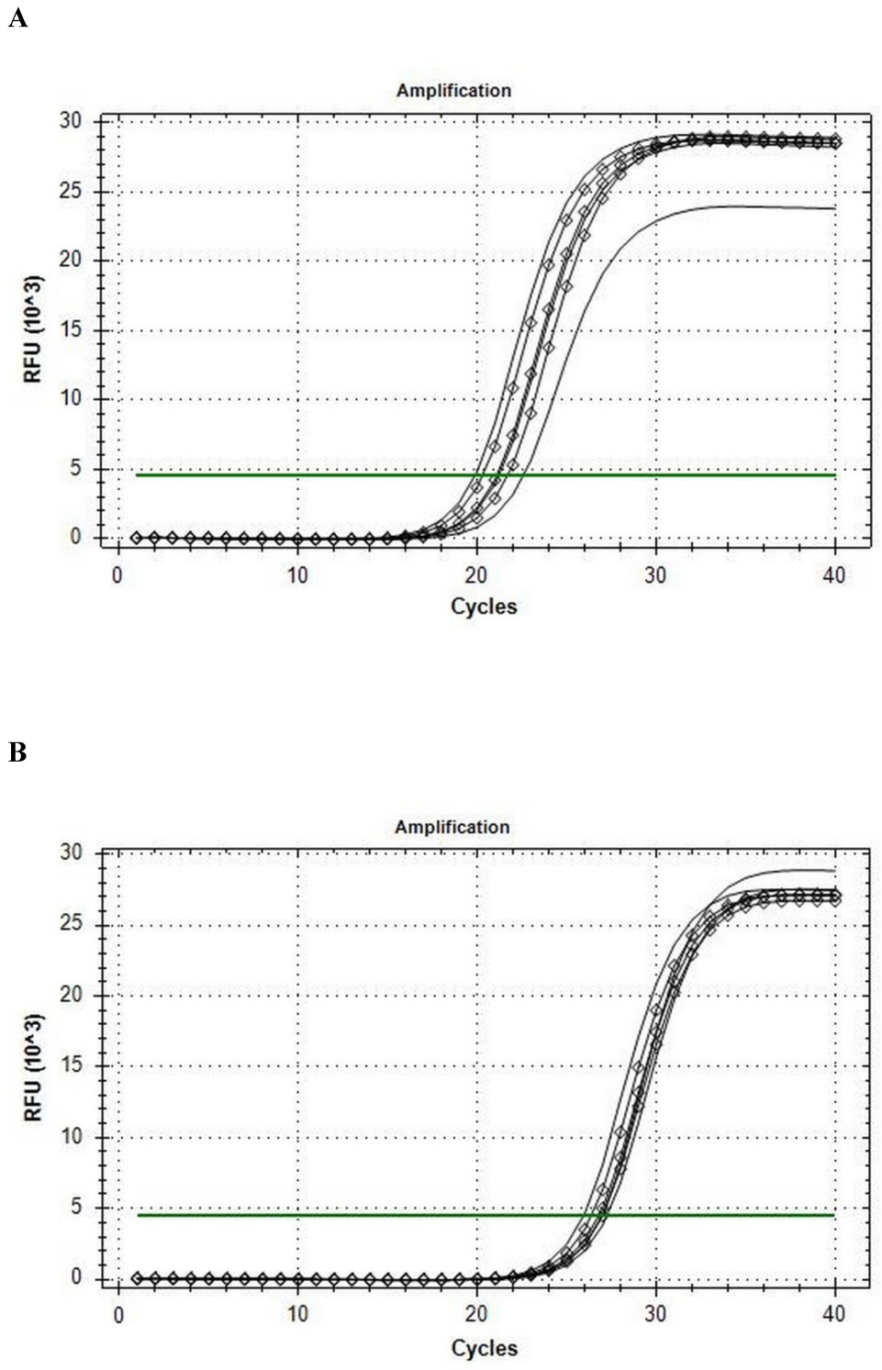
Amplification curves of in rest (line) and post-exercise (diamond) conditions. A, *B2M*; B, *HPRT1.*

The Reference gene panel included the known candidate genes frequently recommended for muscle tissue analysis 30, and the internal PCR performance, reverse transcription, RNA quality, and genomic DNA (gDNA) admixture controls. Fourteen eligible housekeeping genes were tested for comparison in qRT-PCR analysis: *ACTB, B2M, G6PD, GAPDH, GUSB, HMBS, HPRT1, PGK1, RPL13A, RPLPO, RPS18, TBP, TFRC, YWHAZ*. CFX Connect Real-Time PCR System analysis revealed that the *B2M* was the most stable gene enabled for further usage in ddPCR, and that *HPRT1* was the most stable gene during the conditions of our intervention (see Fig. 1). On the base of stability in gene candidates' expression, *B2M* was included into the qRT-PCR and ddPCR analysis of the *BDNF* as a reference gene.

### qRT-PCR *BDNF* polymorphism specific assay

One µl of cDNA solution from the same participant's samples, excluding the female participant, were used in a qRT-PCR with allele-specific *BDNF* assay containing hydrolyzed probes (TaqMan) with both HEX (hexachloro-fluorescein) and FAM (6-carboxyfluorescein) systems of detection for the Val66- and Met66-coding *BDNF* allele's products, respectively (ddPCR Mutation Assay: rs6265, dHsaMDS320493890, BioRad, USA). The *BDNF* allele-specific primers were designed according to Sheikh and colleagues 31. A HEX-based *B2M* Expression Probe Assay (qHsaCPE5053101, BioRad, USA) with the same SsoAdvanced Universal Probes Supermix (BioRad, USA) was ran in parallel and the melting curves were used as a product specificity control. To count the genomic DNA admixture contribution in the mRNA, samples of the participant's total RNA solution were used as a template in concentrations equalized to the residual in cDNA samples. All the reactions were performed in triplicate and the mean was used in subsequent calculations.

Unlike the absolute quantities achieved by ddPCR, reports of FAM and HEX probe detection corresponding to Met66- and Val66-coding BDNF mRNA levels from qRT-PCR could not be compared directly by threshold cycles (C(t)s), probably according to difference in FAM and HEX fluorescence detection sensitivity. Therefore, *BDNF* allele content in genome DNA admixture of RNA samples was used for the mathematical conversion of FAM to HEX detection, then Met66-coding allele's expression was standardized to HEX detection.

PCR program used was: 95°C - 10 min and 40 cycles of 94°C - 30 sec, 55°C - 60 sec with fluorescence detection. The C(t)s - number of cycles that takes to reach threshold - were determined on the HEX and FAM channels by CFX Connect Real-Time PCR System (BioRad, USA). *BDNF* expression levels were calculated by 'delta-delta C(t)' method 32. BDNF C(t)s of each sample was corrected according to the contribution of gDNA admixture using C(t) meanings achieved for the corresponding RNA samples (C(t)_RNA_) by the following formula:

C(t)_correctBDNF_ = C(t)_BDNF_ + log_2_((1 + 2^(C(t)BDNF - C(t)RNA^) / 1)

C(t) for samples where RNA did not give a registered signal were accepted without correction. Corrections of C(t) were performed for FAM and HEX detection independently.

Values of *BDNF* Val66- and Met66-coding alleles present in RNA samples (the alleles' ratio in genome DNA admixture = 1:1) were used as controls for equalizing HEX and FAM detection. The C(t)s of the Met-coding allele were unified to HEX detection afterward in the following formula:

C(t)unifMetBDNF = C(t)_FAM_MetBDNF + (C(t)_HEX_RNA - C(t)_FAM_RNA)

Total *BDNF* expression in heterozygous subjects [C(t) - C(t)_sumBDNF_] was calculated with the formula:

C(t)_sumBDNF_ = C(t)_HEX_BDNF - log_2_((1 + 2^(C(t)HEXBDNF - C(t)unifMetBDNF^) / 1)

### Statistics

Normality of values was determined by a Kolmogorov-Smirnov test. A paired Student's t-test was used to compare condition/changes description and Spearman's rank test for ddPCR to qRT-PCR correlation coefficient in SPSS. An α level of p ≤ 0.05 was used to determine statistical significance. All data are reported as means ± SD.

## Results

Among tested gene candidates as a reference the best stability in the metabolic stressed conditions in muscle was detected for *B2M* (less than 2.5 C(t) range inter- and intra-individually) (Fig. 1a) and *HPRT1* (less than 2 C(t) range inter- and intra-individually) (Fig. 1b) while the least stable for qRT-PCR analysis of stress conditions in the muscle was found to be *GAPDH,* (more than 4 C(t) range inter- and intra-individually). The other analyzed genes also showed low range of variability (less than 3.5 C(t) range inter- and intra-individually) and could possibly be used as a reference gene as well. The results of qRT-PCR Val66Met allele-specific *BDNF* expression analysis with the all mathematical proceeding can be observed in Table 2.

Real-time qPCR analysis detected the relative levels of *BDNF* expression with specificity for the Val66Met polymorphism, according to the absolute quantification performed by ddPCR (see Table 3). The *BDNF* expression showed small variability - often less than 1 C(t) in both the inter-individual differences (K-S(8) = 0.172, p > 0.05) and in regard to testing condition (Rest mean = 1.84 ± 0.79; Stress mean = 0.95 ± 0.34, t(8) = 4.64, p = 0.002) and genotype. Values detected by ddPCR were reported by qRT-PCR with a moderate negative correlation (ρ = - 0.456), suggesting the higher BDNF mRNA copy number, the lower C(t).

## Discussion

BDNF and its gene polymorphism Val66Met have taken on a greater focus of attention regarding its crucial role in neural system development and functioning. Whereas alteration in BDNF production has been suggested to be a marker of neuropathological conditions 3,11,19,33. Investigations examining the effect of exercise on changes in circulating BDNF concentrations have gained much interest as a possible target for the therapy and/or prevention of stress and neurodegeneration-related disorders 22,34,35. Therefore, a precise and accessible method proposed for the laboratory analysis of the allele-specific expression of* BDNF* is a valuable tool for a broader assessment of the *BDNF* Val66Met polymorphism in a clinical and research scenario. By assessing relatively small, but detectable by absolute quantification changes in mRNA levels, we were able to design a protocol for the identification of *BDNF* expression in human tissue regarding to the allele specificity and the metabolic condition by qRT-PCR.

Our results showed that amongst the genes expressing constant patterns (stability) in the human muscle regardless of sex and physiological conditions, the most stable one is the gene of hypoxanthine phosphoribosyl transferase (*HPRT1*). The establishment of a tissue-specific reference gene can help discriminate both distinctive characteristics, and genetic-based changes in cell functioning among different physiological conditions 36,37,38,39;40. Precision in the determination of a gene of reference for comparative analysis of the expression of specific proteins is crucial to enable the assessment of factors that are expressed in low quantities in tissue. These results contribute to the establishment of the *HPRT1* as the most stable gene expressed in muscle tissue, and can be used as reference for comparative analyses of gene expression in humans, regardless of sex and metabolic conditions.

Furthermore, our results indicate that the *GAPDH* (Glyceraldehyde-3-Phosphate Dehydrogenase) gene shows unacceptable parameters (the lowest among the genes tested) as a reference gene for comparisons in different metabolic conditions. Although initially considered a housekeeping gene and widely used as an internal control in experiments on proteins, mRNA, and DNA 41, recent evidence has shown that *GAPDH*, besides having a role in energy metabolism, participates is a more complex regulatory mechanism 42. Nonetheless, the changes in expression patterns of this gene noted during the exercise protocol suggest that *GAPDH* should not be considered as a reference gene when testing different metabolic conditions.

Using the absolute quantification of ddPCR analysis as a reference, we have demonstrated that the identification of the allele-specific expression of *BDNF* by qRT-PCR is feasible. The differential detection of *BDNF* alleles' expression enables the objective analysis of the Val66Met polymorphism role on BDNF functioning, supporting the previous speculations 43,44,45. Moreover, this methodology has proven to be applicable even in small samples, in spite of the small variability showed for the human *BDNF* expression. The qRT-PCR analysis was able to report the results of the ddPCR analysis of *BDNF* expression both in allele- and condition-specific manner (Table 3).

In conclusion, our results indicated that the qRT-PCR protocol was successful in determining the measurement of *BDNF* expression levels in muscle tissue, as well as metabolic stress-delivered and genotype-associated changes in these levels. This protocol appears to be useful for the detection of the Val66Met polymorphism specific *BDNF* expression and can be used in heterogeneous populations.

## Figures and Tables

**Table 1:** Characteristic of Participants in ddPCR™ and qRT-PCR analyses

Subject	Age (years)	BMI (kg/m^2^)	VO_2_max (ml·kg/min)	Genotype
Female	36	19.84	44.00	VAL/VAL
Male	24	21.97	60.00	VAL/VAL
Male	46	22.35	62.00	VAL/VAL
Male	28	21.97	59.38	**VAL/MET**
Male	43	26.31	41.40	VAL/VAL
Male	26	23.10	45.62	**VAL/MET**
Male	22	22.74	51.38	VAL/VAL
Male	46	23.70	56.06	VAL/VAL
Male	28	23.89	61.40	VAL/VAL
Male	36	20.67	54.24	VAL/VAL
Means ± SD	32.9 ± 10.3	23.25 ± 1.4	54.7 ± 7.7	

*VO_2_max, maximal oxygen intake; BMI, body mass index.

**Table 2:** qRT-PCR BDNF analysis. Threshold cycles, C(t)s, calculations.

Genotype	Fluorescense detection	B2Mpre mean	B2Mpost mean	BDNFpre mean	BDNFpost mean	mRNApre mean	mRNApost mean	BDNFpre HEX/FAM correction	BDNFpost HEX/FAM correction	TotalBDNFpre expression in heterozygous	TotalBDNFpost expression in heterozygous
V/V	HEX	24.55	23.26	35.43	34.44	40.11	36.95	35.49	34.67		
	FAM	0	0	0	0	0	0	0	0		
V/V	HEX	21.34	22.46	32.33	34.10	38.22	41.00	32.35	34.11		
	FAM	0	0	0	0	0	0	0	0		
**V/M**	HEX	23.91	24.05	34.21	34.68	36.46	40.30	34.48	34.7	33.86	34.01
	FAM	0	0	29.98	30.83	31.50	35.78	35.37	35.39		
V/V	HEX	26.12	23.60	35.7	33.34	38.62	36.02	35.88	33.54		
	FAM	0	0	0	0	0	0	0	0		
**V/M**	HEX	23.92	23.8	34.42	34.98	36.30	36.56	34.76	35.39	33.96	34.66
	FAM	0	0	30.09	31.01	31.62	32.13	35.19	35.99		
V/V	HEX	23.21	23.17	32.29	33.37	35.29	35.63	32.45	33.64		
	FAM	0	0	0	0	0	0	0	0		
V/V	HEX	24.37	24.61	34.04	34.23	36.94	37.71	34.22	34.35		
	FAM	0	0	0	0	0	0	0	0		
V/V	HEX	24.6	22.95	33.19	32.19	36.65	36.65	33.32	32.25		
	FAM	0	0	0	0	0	0	0	0		

**Pre**, Analyses from the rest condition; **Post**, Analyses from after effort test

**Table 3:** qRT-PCR results validation by ddPCR. Condition- and allele-specific *BDNF* expression differences.

Genotype	qRT-PCR			ddPCR			qRT-PCR		ddPCR	
	Tpre BDNF-B2M	Tpost BDNF-B2M	T BDNF post /pre	Tpre BDNF /1000B2M	Tpost BDNF /1000B2M	T BDNF post /pre	Pre MetBDNF /ValBDNF	Post MetBDNF /ValBDNF	Pre MetBDNF /ValBDNF	Post MetBDNF /ValBDNF
V/V	10.94	11.41	0.721	1.444	0.703	0.487				
V/V	11.01	11.66	0.641	1.792	1.17	0.653				
**V/M**	9.950	9.960	0.996	1.427	0.926	0.649	0.541	0.621	0.707	0.614
V/V	9.760	9.950	0.877	0.793	0.498	0.628				
**V/M**	10.04	10.86	0.566	1.524	0.910	0.598	0.741	0.662	0.660	0.968
V/V	9.250	10.48	0.427	2.319	0.745	0.321				
V/V	9.850	9.740	1.077	2.045	1.110	0.543				
V/V	8.720	9.300	0.665	3.460	1.607	0.464				
Means ± SD	T BDNF post/pre, 0.736 ± 0.205			T BDNF post/pre, 0.543 ± 0.115			MetBDNF/ValBDNF, 0.641 ± 0.084		MetBDNF/ValBDNF, 0.737 ± 0.158	

**Pre**, Analyses from the rest condition; **Post**, Analyses from exercise protocol; Tpre and Tpost - total (both alleles) *BDNF* expression, ValBDNF, MetBDNF - allele-specific *BDNF* expression.
